# Evaluation of Indigo Naturalis Prepared Using a Novel Method: Anti‐Inflammatory Activities Against Colonic Cancer Cell Lines

**DOI:** 10.1155/mi/1141244

**Published:** 2026-02-03

**Authors:** Xianxiang Xu, Lin Lin, Wenjie Ning, Xinyi Zhou, Aftab Ullah, Xiaoping Huang, Huiyong Yang, Xunxun Wu, Yong Diao

**Affiliations:** ^1^ School of Medicine, Huaqiao University, 269 Chenghua North Road, Quanzhou, 362021, China, hqu.edu.cn

**Keywords:** aryl hydrocarbon receptor (AhR), indigo naturalis prepared using a novel method (NIN), inflammation-cancer transformation, ulcerative colitis

## Abstract

Ulcerative colitis (UC) is a chronic inflammatory bowel disease (IBD), and cancerous transformation of UC is closely associated with chronic inflammation of colonic tissues. Indigo naturalis (IN) prepared using a novel method (NIN) has exhibited beneficial efficacy against inflammatory and cancerous colonic cells. However, the underlying mechanism still remains to be elucidated. This study aimed to construct an inflammation model by the HT‐29 colonic cancer cell line and to investigate the effect of NIN on an inflammatory cancer state and the possible mechanism. The results showed that NIN could reduce the increased expression of pro‐inflammatory cytokine IL‐1β in the inflammatory cancer cells and attenuate the inflammatory response; elevate the low expression of MUC2 in the inflammatory cancer state and restore the mucin secretion function; and inhibit the proliferation of HT‐29 cells. Based on the activation of the aryl hydrocarbon receptor (AhR) signaling pathway, NIN increases the expression of the Wnt/β‐catenin signaling pathway inhibitor Rnf43, inhibits the expression of nonphosphorylated β‐catenin, and reduces the level of the pathway downstream target gene Axin2, which in turn inhibits the Wnt/β‐catenin signaling pathway and inhibits the expression of Lgr5, a stem cell gene of colorectal cancer (CAC). The production of the above effects of NIN was blocked by the AhR antagonist CH223191. The in vitro studies verified that NIN alleviated UC by activing AhR signaling pathway, which in turn inhibited the Wnt/β‐catenin signaling pathway. The possible mechanism of NIN on UC could be explained starting from the inflammation‐cancer transformation. Furthermore, comprehensive research is expected between inflammation and cancer development.

## 1. Introduction

Ulcerative colitis (UC) is an inflammatory disease of the gastrointestinal tract, which is characterized with a wide range of lesions, a tendency to recur, and a tendency to become cancerous [1, 2]. Transformation of UC to cancer is strongly associated with chronic inflammation in colonic tissues, and patients with chronic UC are at a higher risk of developing colorectal cancer (CAC)[3, 4]. Inflammatory stress at the site, and the presence of intestinal microbiota and cytokines contribute to the inflammatory bowel disease (IBD) and colitis‐associated CAC [5, 6]. The patients face an increased risk of developing CAC. In Asian UC patients, malignant transformation typically begins 10–20 years post‐diagnosis, whereas in North American CAC patients, the risk significantly escalates after 30 years [7]. Effectively managing chronic inflammation and mucosal damage in UC is vital to mitigate the risk of cancerous transformation [8]. Despite advances, controlling this progression remains a significant challenge in UC treatment [9].

Traditional Chinese medicine (TCM) has shown great progress in the research of treating UC [10]. Indigo naturalis (IN), also called “Qingdai,” is a commonly used TCM prepared from the processed leaves or stems of *Baphicacanthus cusia* (Nees) Bremek and other plants [11]. IN has been found to possess anti‐inflammatory, antioxidant, antibacterial, and immunomodulatory effects [12–14]. Its active ingredients, indirubin and tryptophan, have shown significant inhibitory effects on a variety of cancers [15, 16]. The antitumor ability, mainly from indirubin, materialize IN managing acute promyelocytic leukemia (APL) in some Chinese medicinal formulations [17]. Contemporary research has expanded the applications of IN, revealing its potential in treating liver damage, nephritis, oral ulcers, and various skin disorders [18–20]. However, most of the current studies on these diseases are at the basic research stage, and more data support from clinical studies is still needed. More interestingly, indole compounds such as indirubin and indigo, the active ingredients of IN, are endogenous ligands for the aryl hydrocarbon receptor (AhR) [21] which is involved in the proliferation of a wide range of cells leading to intestinal inflammation and tumors. The binding of AhR to the ligands may be involved in the regulation of inflammatory pathways [22], tumorigenesis [23], and immune responses [24] in the intestinal tract. IN, along with its active ingredients indirubin and indigo, was found to have the ability to regulate IL‐10 and IL‐22 expression via agonism of AhR, which in turn regulates the mucosal barrier function and consequently improves the symptoms of UC in experimental mice induced by dextran sodium sulfate (DSS) [25].

Recently, indirubin in IN has got more attention due to its significant apoptotic effects in a variety of tumor cells [26, 27]. Although IN‐based Chinese herbal preparations have achieved good efficacy in the clinical treatment of UC, the mechanism of IN’s role in UC‐associated inflammation‐cancer transformation is still not clear. The conventional method of concoction often leads to the excessive use of lime and results in a formulation with a low concentration of active ingredients. We have innovatively advanced the traditional IN concoction process, significantly enhancing the production yields of indigo and indirubin. NIN refers to IN prepared using a novel method. In DSS salt induced UC mice, NIN can significantly improve the symptoms of colitis, reduce colonic tissue damage, improve inflammatory response, and promote mucosal repair. The anti‐UC effect of NIN was related to the agonism of AhR [28]. Based on the existing reports on the antitumor effects of indirubin, we hypothesized that NIN alleviates UC by activating AhR, subsequently disrupting the inflammatory‐cancer transformation by modulating interactions within various signaling pathways. Conclusively, our study explores the therapeutic potential and underlying mechanisms of these formulations in mitigating UC through its inflammation‐cancer nexus.

## 2. Materials and Methods

### 2.1. Reagents

HT‐29 was purchased from ATCC Cell Bank, HK‐2 was purchased from Pronosay Life Sciences. LPS and DMSO were purchased from Solebo. RPMI‐1640 and DMEM/F12 medium were purchased from Gibco. Penicillin–streptomycin solution (100×), Trypticase cell digestion solution (0.25%, containing phenol red), RNAeasy Animal RNA Extraction Kits, RIPA Lysis Buffer, protease phosphatase inhibitor mixture, BCA protein concentration measurement kits, SDS‐PAGE protein supersampling buffer (5×), SDS‐PAGE gel rapid preparation kits, QuickBlock western primary antibody dilution solution, QuickBlock Western Secondary Antibody Dilution Solution, Western wash solution (10×), β‐actin Rabbit mAb, and HRP‐labeled goat Anti‐rabbit IgG (H + L), were all purchased from Biyun Tian. AhR (D5S6H) Rabbit mAb, non‐phospho (active) β‐catenin (Ser 33/37/Thr 41) (D13A1) Rabbit mAb were purchased from CST. HiScript Q RT SuperMix for qPCR (+gDNA wiper), ChamQ Universal SYBR qPCR Master Mix were purchased from Novozymes. CH223191 was purchased from MCE. Serum‐free cell cryopreservative was purchased from Sinsaime. PVDF membrane (0.45 μm) was purchased from Millipore.

### 2.2. Main Reagent Preparation

NIN was synthesized using our laboratory‐developed concoction process [21]. NIN is powder with the characters of blue–black color, light quality, easy flying, mild odor, and flavor, shown in Figure 1. The percentages of indirubin and indigo in SIN were 0.33% and 2.85% quantified by HPLC method. 160 mg NIN was dissolved in 1 mL DMSO, after which 1 mL RPMI 1640 or DMEM/F12 medium was added depending on the cell type involved. This solution was then thoroughly mixed, filtered to eliminate bacteria, resulting in an 80 mg/mL NIN stock solution. For the CH223191 test solution, 1.50 mL DMSO was used to dissolve the CH223191 following the product specifications, then filtered to obtain a 10 mM CH223191 stock solution. Last, 2 mg LPS was mixed with 4 mL RPMI 1640 medium, filtered to create a 0.5 mg/mL LPS stock solution.

Figure 1Character and identification of NIN (a) character of NIN. (b) Thin‐layer chromatography identification: 1‐SIN; 2, 3‐NIN; 4‐Indigo; 5‐Indirubin.

**Figure:**
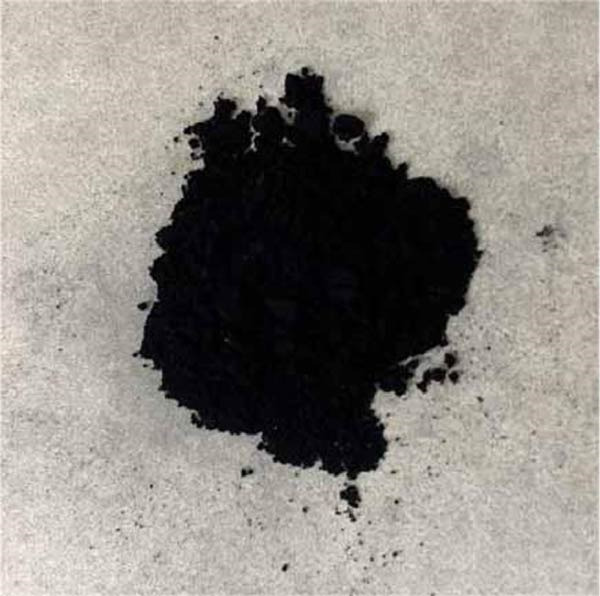
(a)

**Figure:**
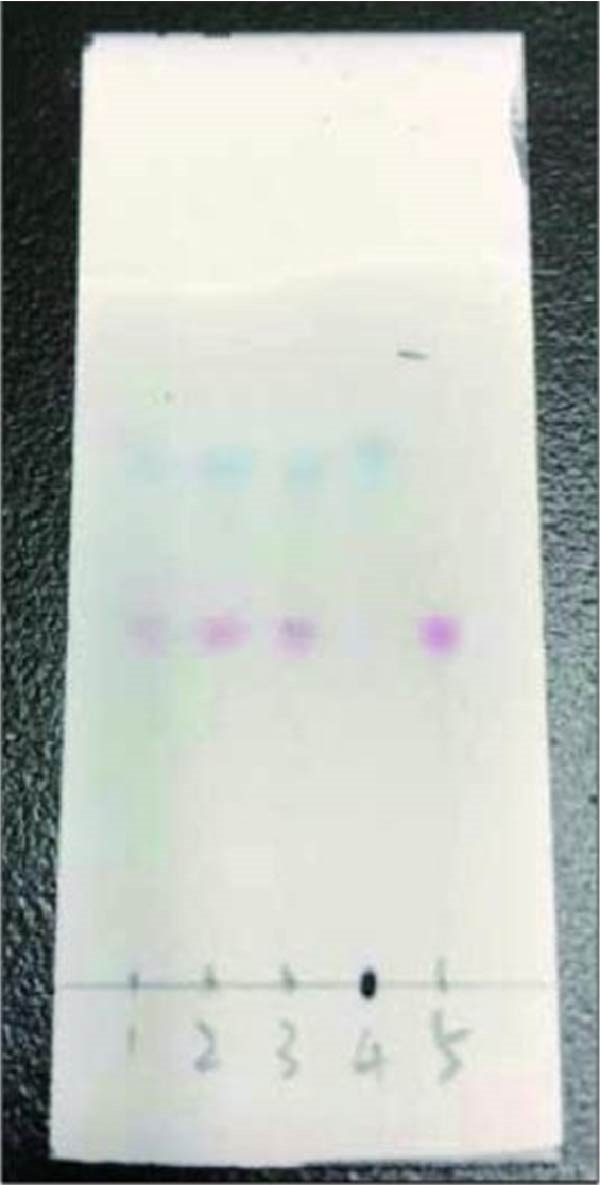
(b)

### 2.3. Cell Viability Assay

Serum‐free DMEM/F12 basal medium was used to prepare five concentration gradients of NIN test solution: 0, 50, 100, 200, and 400 μg/mL. Serum‐free RPMI 1640 basal medium was used to prepare different concentration gradients of NIN test solution: 0, 50, 100, and 200 μg/mL. Three distinct groups were established for the experiment: a blank group, a control group, and a test group. The blank group received no cell inoculation; instead, an equivalent volume of basal medium was added on the drug administration day. In the control group, cells were inoculated, and the basal medium included DMSO but no drug on the drug administration day. Conversely, the test group involved cell inoculation followed by the addition of varying concentrations of the drug solution on the same day.

HK‐2/HT‐29 cells in the logarithmic growth phase were seeded into 96‐well plates at a density of 5000 cells per well, with 100 μL of culture medium per well. After 24 h of incubation, the initial medium was replaced with 100 μL of the designated solution according to the group setup. Subsequent drug treatments were carried out over periods of 24, 48, and 72 h. Posttreatment, a CCK‐8 solution was added, and after 2 h of incubation, the absorbance at 450 nm was measured using an enzyme‐linked assay. Cell viability was then calculated using the following formula:

Cell viability=Absorbance value of experimental group−absorbance value of blank group/absorbance value of control group−absorbance value of blank group×100%.

### 2.4. Cell Culture and Processing

The NIN stock solution was diluted to concentrations of 100, 200, and 400 μg/mL using serum‐free RPMI 1640 basal medium. On the dosing day, 1 mL NIN solution was combined with medium to achieve final concentrations of 50, 100, and 200 μg/mL. The CH223191 stock solution was similarly diluted to 40 μM on the day of administration to achieve a 10 μM concentration. Furthermore, based on preliminary results, 120 μL of a 0.5 mg/mL LPS stock solution was diluted to 30 μg/mL on the administration day.

The control group, model group, and three drug concentrations were established for both the test and antagonist groups. HT‐29 cells were seeded at 1 × 106 cells/well into six‐well plates for 24 h. After removing the original medium and washing thrice with PBS, the cells were treated with the respective drug solutions for another 24 h, as outlined in Table 1.

**Table 1:** Breakdown of drugs administered in each group.

Groups	1640 Basic (μL)	LPS (μL)	NIN (μL)	CH223191 (μL)	DMSO (μL)
CON	1997.5	—	—	—	2.5
MOD	1877.5	120	—	—	2.5
50	880	120	1000	—	—
100	880	120	1000	—	—
200	880	120	1000	—	—
50 + CH223191	380	120	1000	500	—
100 + CH223191	380	120	1000	500	—
200 + CH223191	380	120	1000	500	—

### 2.5. qRT‐PCR Assay

Total RNA was extracted using the RNA extraction kits. The RNA concentration was determined with a microspectrophotometer. RNA purification and reverse transcription were carried out using the HiScript Q RT SuperMix. The cDNA was prepared in accordance with ChamQ Universal SYBR qPCR Master Mix protocols. The primers were synthesized by Sangon Biotech (Shanghai), as shown in Table 2. The PCR reaction system included 10 μL 2× ChamQ Universal SYBR qPCR Master Mix, 0.4 μL each primer (10 μM), and refilled ddH_2_O to 20 μL. PCR reaction was initiated by 30 s denaturation at 95°C, followed by 40 cycles of 95°C for 10 s, 60°C for 30 s, and final elongation at 60°C for 1 min. The results were quantified using the 2^−ΔΔCT^ method. Every step was taken in accordance with the manufacturer’s guidelines.

**Table 2:** Breakdown of primers for real‐time PCR.

Gene primer name	Gene primer name	Sequences (5’–3’)
*GAPDH*	Forward	TATCGTGGAAGGACTCAT
	Reverse	GGATGATGTTCTGGAGAG
*IL-1β*	Forward	GGATATGGAGCAACAAGT
	Reverse	CAGGACAGGTACAGATTC
*Muc2*	Forward	TCCTCTACCTCCATCAATA
	Reverse	CCAATCAATTCTGTGTCTC
*Cyp1a1*	Forward	CCTATTCTTCGCTACCTAC
	Reverse	CTCCTTGACCATCTTCTG
*AhR*	Forward	TAACCCAGACCAGATTCCTCCAGA
	Reverse	CCCTTGGAAATTCATTGCCAGA
*Lgr5*	Forward	AACACTGACTCTGAATGG
	Reverse	GCACTTGGAGATTAGGTAA
*Rnf43*	Forward	AATAACTCCAGCAGAAGG
	Reverse	CAGATTGTCGTCATCACT
*Axin-2*	Forward	CCACCAAGACCTACATAAG
	Reverse	CCACTCCTCACATATTCG

### 2.6. Western Blotting Assay

After 24 h’ treatment, the cells were lysed for protein extraction using RIPA buffer. Protein concentrations were quantified using the BCA assay. In total, 20 μg of denatured proteins were added and processed through SDS‐PAGE and subsequently blotted onto a polyvinylidene difluoride membrane in cold methanol‐containing blotting buffer. The membrane was blocked in QuickBlock Western blocking buffer for 15 min and then incubated overnight at 4°C with the primary antibody as shown in Table 3. After incubation, it was washed three times with TBST and incubated with the secondary antibody at room temperature for 1 h. The membrane was then exposed to freshly prepared ECL solution and visualized with a gel imaging system. β‐actin served as the internal control. Densitometry of the blot was analyzed using ImageJ software 1.8.0.

**Table 3:** Breakdown of western blotting assay staining antibodies.

Antibody name	Dilution times	Antibody source (stock number)
AhR (D5S6H) Rabbit mAb	1:1000	CST (#83200)
Non‐phospho (active) β‐catenin (Ser33/37/Thr41) (D13A1) Rabbit mAb	1:1000	CST (#8814)
β‐actin rabbit monoclonal antibody	1:1000	Beyotime (AF5003)
Horseradish peroxidase labeled goat anti‐rabbit IgG (H + L)	1:50	Beyotime (A0208)

### 2.7. Statistical Analysis

Graphpad Prism 6 is used for drawing graphs. All data were presented as mean ± standard deviation. The statistical analysis was conducted with SPSS 18.0, in which one‐way ANOVA was employed to compare the significance levels. All experiments were conducted independently five times.

## 3. Results

### 3.1. Effect of NIN on HK‐2 Cell Viability

**Figure 2:**
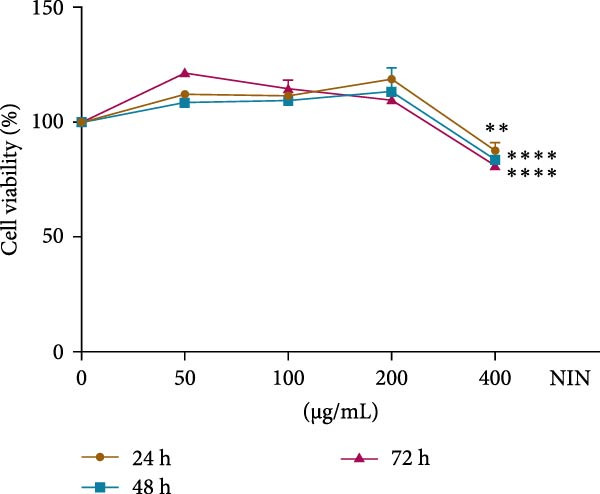
Effect of NIN on HK‐2 cell viability. *n* = 5,  ^∗∗^ *p* < 0.01,  ^∗∗∗∗^ *p* < 0.0001 vs. the normal control.

In order to determine the safe dose of NIN in normal cells, five concentration gradients of NIN were chosen to act on the HK‐2 normal human renal tubular epithelial cell line for 24 h, 48 h, and 72 h, respectively, and the cell viability was calculated. The results showed that the cell viability at NIN concentrations of 0–200 μg/mL was comparable to that at the drug concentration of 0. When the concentration reached 400 μg/mL, the cell viability decreased very significantly, so the safe dose of NIN was screened to be 0–200 μg/mL (Figure 2).

### 3.2. NIN Decreases the Expression of Inflammatory Factor IL‐1*β* in HT‐29 Cells

To investigate the effect of NIN on the level of inflammation in the inflammatory cancer, we examined the expression level of the pro‐inflammatory cytokine IL‐1β mRNA. The results showed that 100 μg/mL NIN reduced the IL‐1β expression level compared with MOD (Figure 3a).

Figure 3Effect of NIN on inflammatory factor levels in inflammatory HT‐29 cells. (a): on IL‐1β expression. (b): with the AhR antagonist. *n* = 5,  ^∗∗^ *p* < 0.01 vs. the normal control. ^#^ *p* < 0.05 vs. the model.

**Figure:**
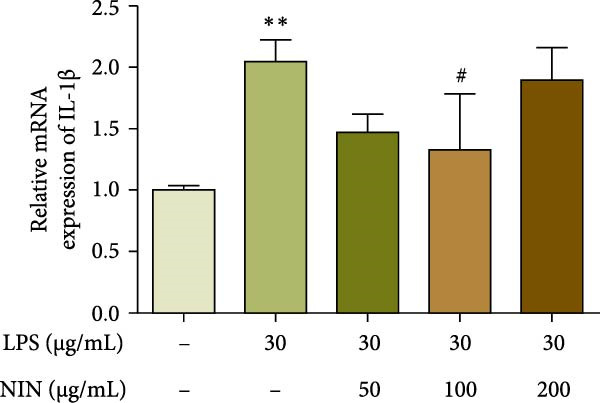
(a)

**Figure:**
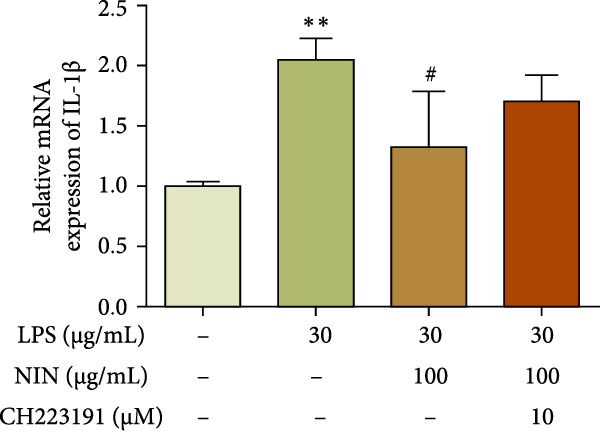
(b)

To investigate whether the reduction of IL‐1β expression by 100 μg/mL NIN acted through the agonism of AhR, we chose CH223191, an antagonist of AhR, to act on the 100 μg/mL NIN group and detected the expression of IL‐1β mRNA. The results showed that the IL‐1β level was no longer statistically different in the 100 μg/mL NIN group with the addition of CH223191 compared to the MOD group and that the effect of 100 μg/mL NIN to reduce the IL‐1β level was blocked by CH223191 (Figure 3b).

### 3.3. NIN Elevates the Low Expression of Muc2 in HT‐29 Cells

Here we explored the effect of NIN on the expression level of Muc2 mRNA in cells under inflammatory cancer. The results showed that 200 μg/mL NIN could increase the Muc2 mRNA level in inflammatory cancer cells significantly (Figure 4a). To investigate whether 200 μg/mL NIN reduced Muc2 expression by agonizing AhR, we chose an antagonist of AhR to act in the 200 μg/mL NIN group and detected the expression of Muc2 mRNA. Compared with the 200 μg/mL NIN group, the 200 μg/mL NIN group with the addition of CH223191 changed the statistical difference in Muc2 levels compared with the MOD group, and the difference in Muc2 levels between the two groups was highly significant when compared with the 200 μg/mL NIN group alone, and the effect of the 200 μg/mL NIN to increase the Muc2 levels was blocked by CH223191 (Figure 4b).

Figure 4Effect of NIN on Muc2 levels in inflammatory HT‐29 cells. (a): on Muc2 mRNA expression. (b): with the AhR antagonist. *n* = 5, ^##^ *p* < 0.01, ^####^ *p* < 0.0001 vs. the model. ^$$$$^ *p* < 0.0001 vs. the AhR antagonist.

**Figure:**
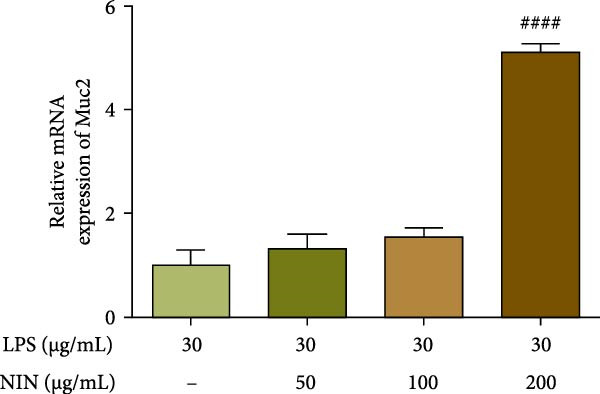
(a)

**Figure:**
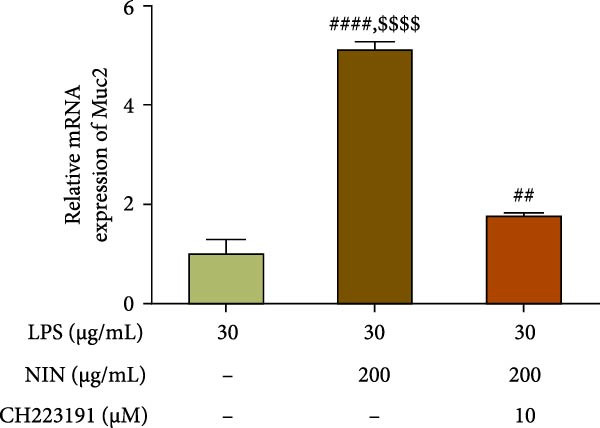
(b)

### 3.4. NIN Inhibits HT‐29 Cell Proliferation

**Figure 5:**
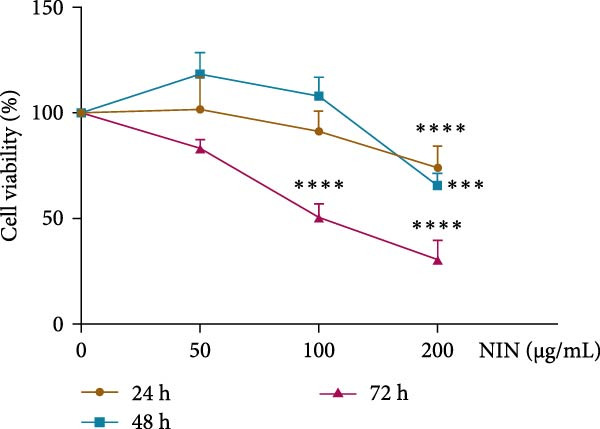
Effect of NIN on proliferation of HT‐29 cells. *n* = 5,  ^∗∗∗^ *p* < 0.001,  ^∗∗∗∗^ *p* < 0.0001 vs. the normal control.

Based on the reported anticancer properties of indirubin, the effect of NIN was explored within a safe dosage range on the HT‐29 human colon cancer cell line over 24, 48, and 72 h. Results demonstrated a notable reduction in cell viability at 72 h with 100 μg/mL NIN compared to the control (0 μg/mL). Further reduction was observed when the NIN concentration was increased to 200 μg/mL, underscoring its potential to inhibit tumor cell proliferation effectively. These findings suggest that NIN, at these concentrations, effectively suppresses the growth of cancer cells (Figure 5).

### 3.5. NIN Inhibits Lgr5 Levels in Inflammatory HT‐29 Cells

Our previous study (not yet published) found that 100 and 200 μg/mL NIN inhibited the proliferation of tumor cells, to further investigate the antitumor effect of NIN on inflammatory cancer cells, Lgr5, a colon cancer stem cell gene, was selected as an evaluation index and its expression level was detected. The results showed that 100 μg/mL NIN could reduce the expression level of Lgr5, and 200 μg/mL NIN could significantly reduce the expression level of Lgr5 (Figure 6a).

Figure 6Effect of NIN on the level of Lgr5 in inflammatory HT‐29 cells. (a): on Lgr5 mRNA expression. (b): with the AhR antagonist. n = 5, ^#^ *p* < 0.05, ^###^ *p* < 0.001 vs. the model.

**Figure:**
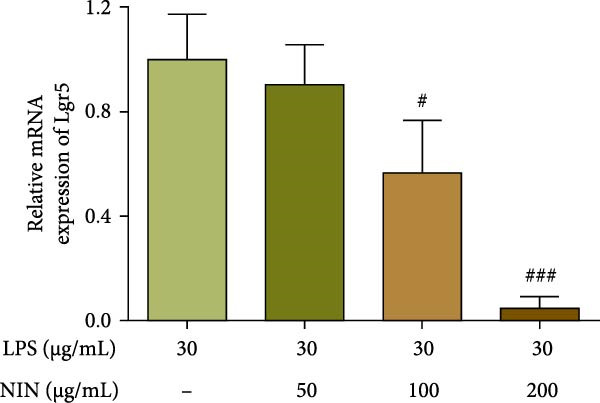
(a)

**Figure:**
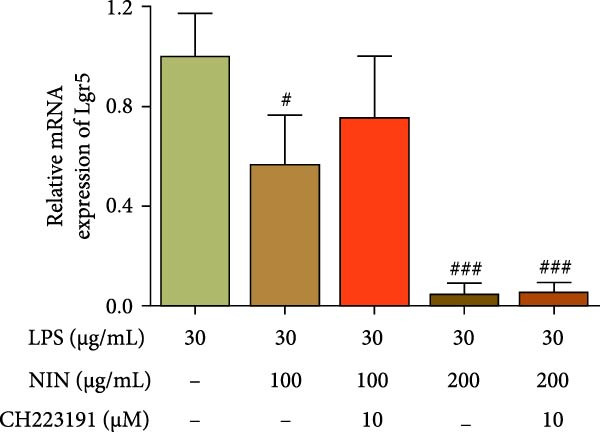
(b)

To investigate whether the reduction of Lgr5 expression by 100 and 200 μg/mL NIN acted through the agonism of AhR, we chose the antagonist to treat the cells and then detected the expression of Lgr5 mRNA. The results showed that there was no longer a statistically significant difference in Lgr5 levels between the 100 μg/mL NIN group, and the MOD group with the addition of CH223191, and the effect of 100 μg/mL NIN in reducing IL‐1β levels was blocked by CH223191; whereas for the 200 μg/mL NIN group, there was no significant effect of the addition of the antagonist on the expression of Lgr5 (Figure 6b).

### 3.6. Effect of NIN on AhR Signaling Pathway in Inflammatory HT‐29 Cells

In this study, we examined the influence of NIN on the Cyp1a1 mRNA expression within inflammatory and cancer cells, a downstream target of AhR signaling pathway. Our findings revealed that concentrations of NIN at 50, 100, and 200 µg/mL significantly elevated the expression levels of Cyp1a1 mRNA. Notably, the effectiveness of NIN in enhancing Cyp1a1 expression initially increased with higher concentrations but subsequently diminished (Figure 7a). To assess if NIN’s upregulation of Cyp1a1 was mediated through activating AhR, we treated the cells with the AhR antagonists at these concentrations and monitored the expression of Cyp1a1 mRNA. The results indicated no significant change in Cyp1a1 expression between the NIN‐treated groups at 50 and 100 µg/mL with the antagonist and the control, whereas a highly significant difference was observed without the antagonist, confirming that the effect was blocked by CH223191. However, at 200 µg/mL, the antagonist markedly inhibited the NIN‐induced increase in Cyp1a1 expression levels (Figure 7b).

Figure 7Effect of NIN on AhR signaling pathway in inflammatory HT‐29 cells. (a): on Cyp1a1 mRNA expression. (b): with the AhR antagonist. (c): on AhR mRNA expression. (d): with the AhR antagonist. *n* = 5, ^#^ *p* < 0.05, ^##^ *p* < 0.01, ^####^ *p* < 0.0001 vs. the model. ^$$^ *p* < 0.01, ^$$$$^ *p* < 0.0001 vs. the AhR antagonist.

**Figure:**
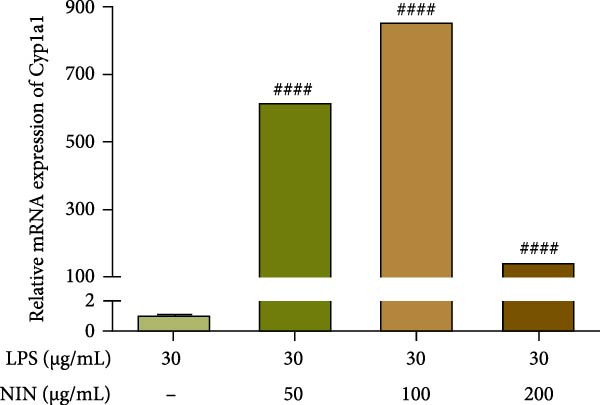
(a)

**Figure:**
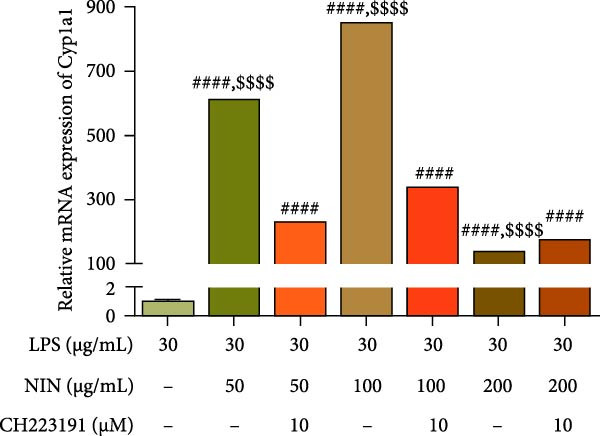
(b)

**Figure:**
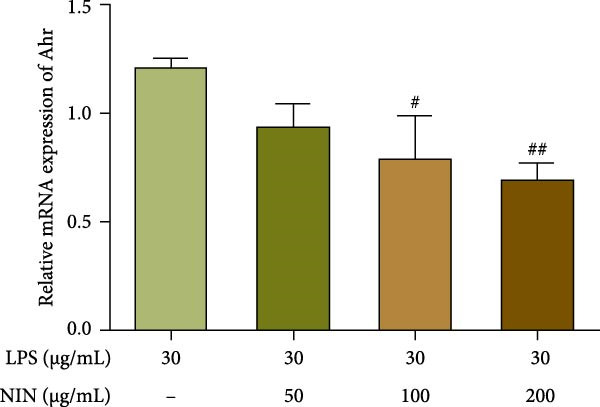
(c)

**Figure:**
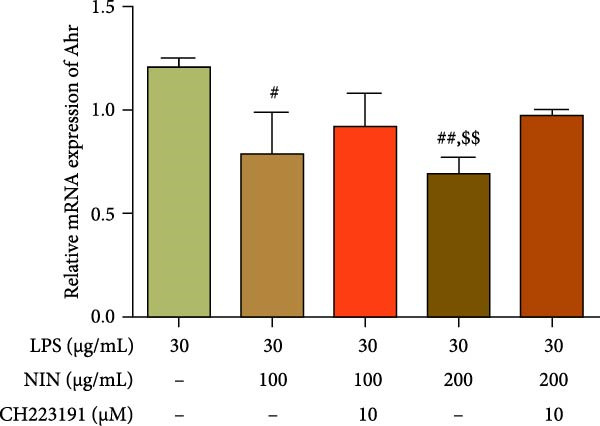
(d)

Furthermore, we explored the impact of NIN on AhR mRNA levels, another crucial component of the AhR signaling pathway. The findings demonstrated that NIN at concentrations of 100 and 200 µg/mL substantially decreased AhR mRNA levels in these cells (Figure 7c). Posttreatment with the AhR antagonist CH223191, the notable reduction in AhR expression by NIN was effectively nullified, indicating that the decrease was AhR‐dependent (Figure 7d).

**Figure 8:**
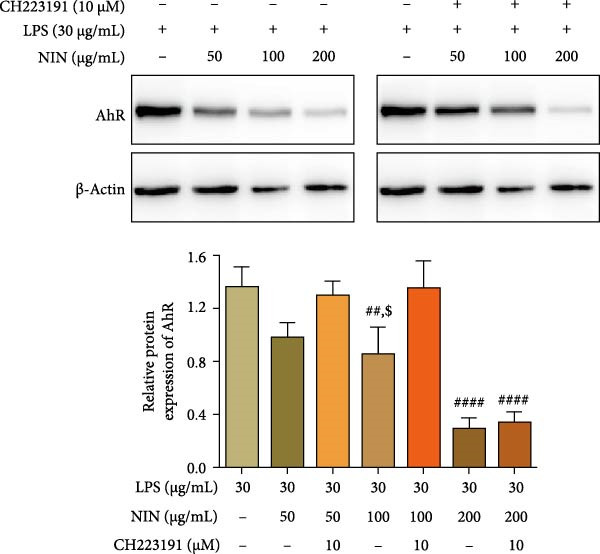
Effect of NIN on AhR protein expression level in AhR signaling pathway in inflammatory HT‐29 cells. Western blot method was used to detect the protein levels of AhR. β‐actin was used as an internal control. *n* = 5, ^##^ *p* < 0.01, ^####^ *p* < 0.0001 vs. the model. ^$^ *p* < 0.05 vs. the AhR antagonist.

The study further extended to evaluate the effect of NIN on AhR protein expression. Compared to the control group, a significant reduction in AhR protein levels was observed with 100 µg/mL NIN, which was completely reversed by CH223191. In contrast, at 200 µg/mL, the antagonist did not significantly alter the AhR expression profile, suggesting a different modulation mechanism at higher concentrations (Figure 8).

### 3.7. Effect of NIN on Wnt/β‐Catenin Signaling Pathway in Inflammatory HT‐29 Cells

This study aimed to explore the impact of nigrin (NIN) on the mRNA expression levels of ring finger protein Rnf43, an inhibitor of the Wnt/β‐catenin signaling pathway, in HT‐29 cells under conditions of inflammatory cancer. The results indicated that 50, 100, and 200 μg/mL NIN significantly enhanced the expression of Rnf43 mRNA in a dose‐dependent manner, as depicted in Figure 9a. Further investigations were conducted to determine whether this increase in Rnf43 expression was mediated through activing AhR. Treatment with the AhR antagonist CH223191 altered the expression outcomes. In the presence of 50 μg/mL NIN and CH223191, the increase of Rnf43 was inhibited. This blocking effect was also observed at higher NIN concentrations (100 and 200 μg/mL), which significantly reduced Rnf43 expression compared to the control, as shown in Figure 9b.

Figure 9Effect of NIN on Wnt‐β‐catenin signaling pathway in inflammatory HT‐29 cells. (a): on Rnf43 mRNA expression. (b): with the AhR antagonist. (c): on Axin2 mRNA expression. (d): with the AhR antagonist. *n* = 5, ^#^ *p* < 0.05, ^##^ *p* < 0.01, ^###^ *p* < 0.001 vs. the model. ^$^ *p* < 0.05, ^$$^ *p* < 0.01 vs. the AhR antagonist.

**Figure:**
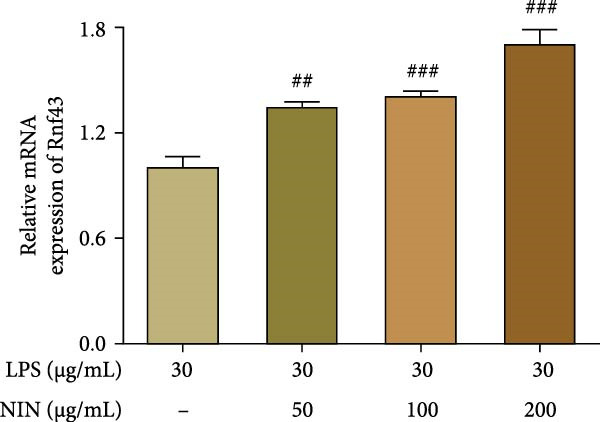
(a)

**Figure:**
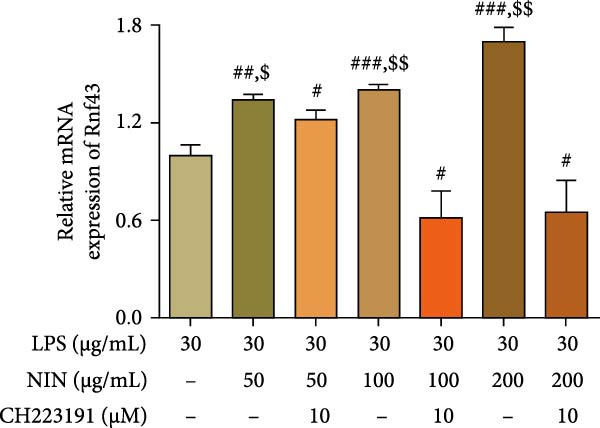
(b)

**Figure:**
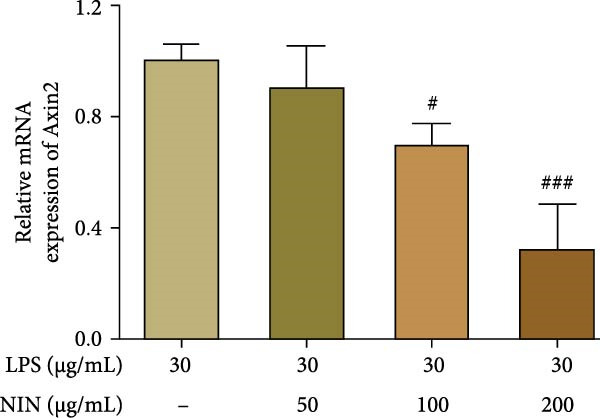
(c)

**Figure:**
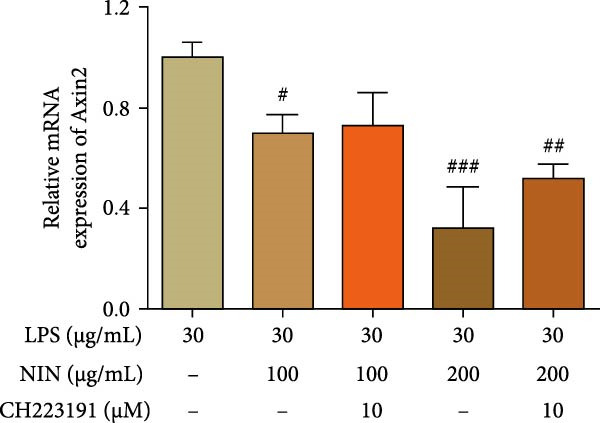
(d)

In addition to Rnf43, an inhibitor of the Wnt/β‐catenin signaling pathway, the effect of NIN on the expression of Axin2 mRNA, a target gene downstream of the AhR signaling pathway, was investigated in inflammatory cancer cells, and the results showed that 100 and 200 μg/mL NIN could significantly reduce the level of Axin2 mRNA in inflammatory cancer cells (Figure 9c). To investigate whether the reduction of Axin2 expression by 100, 200 μg/mL NIN acted through the agonism of AhR, the expression of Axin2 mRNA was detected after treating the cells with AhR antagonist, and the results were as shown in Figure 9d, the statistical difference of Axin2 level in the group with the addition of CH223191 of 100, 200 μg/mL NIN group was statistically different from that in the group with the addition of MOD changed, the 100 μg/mL NIN group was no longer statistically different from the MOD group, and the statistical difference in the 200 μg/mL NIN group was reduced to *p*  < 0.01, indicating that the effect of 100 and 200 μg/mL NIN in reducing Axin2 levels was blocked by CH223191.

**Figure 10:**
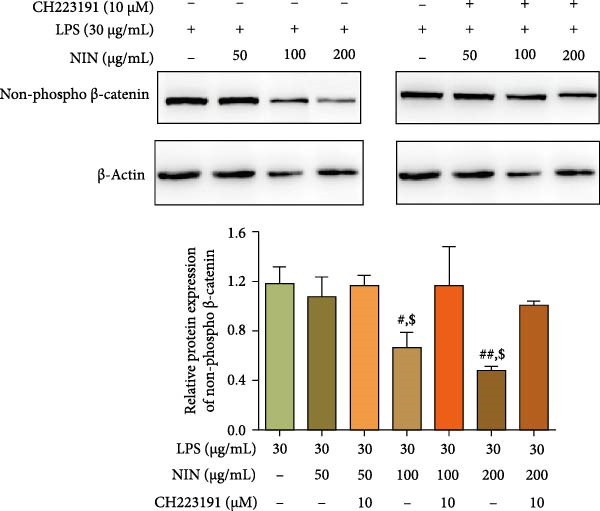
Effect of NIN on the expression level of non‐phospho β‐catenin protein in the Wnt/β‐catenin signaling pathway in inflammatory HT‐29 cells. Western blot method was used to detect the protein levels of β‐catenin. β‐actin was used as an internal control. *n* = 5, ^#^ *p* < 0.05, ^##^ *p* < 0.01 vs. the model. ^$^ *p* < 0.05 vs. the AhR antagonist.

Based on the previous investigation of NIN on the expression of Rnf43 and Axin2 mRNA in the Wnt/β‐catenin signaling pathway, the present experiment was conducted to investigate the effect of NIN on the expression of the key protein, non‐phosphorylated β‐catenin (non‐phospho β‐catenin), in inflammatory carcinoma cells. As shown in Figure 10, 100 μg/mL NIN reduced the expression level of non‐phosphor β‐catenin protein, and 200 μg/mL NIN significantly reduced the expression level of non‐phosphor β‐catenin protein, as compared with MOD. After the addition of an antagonist, the expression of non‐phosphor β‐catenin in the 100 and 200 μg/mL NIN group was no longer statistically different compared with MOD, and the effect of 100 and 200 μg/mL NIN in decreasing the expression level of non‐phosphor β‐catenin protein was blocked by CH223191.

## 4. Discussion

UC undergoes repeated inflammatory stress, which may cause genetic alterations and lead to cancer [29]. Therefore, controlling chronic inflammation and mucosal damage is crucial in the inflammation‐cancer transformation of UC. Given the carcinogenic potential of UC and the significant indirubin levels in NIN, alongside its noted anti‐inflammatory and antitumor properties, this study initially determined a safe dosage of NIN between 0 and 200 μg/mL. Subsequently, it assessed the effects on inflammation, mucin secretion, and tumor cell proliferation in colon cells affected by inflammation and cancer within this range.

We used the HT‐29 colon cell line to create an inflammation model. The inflammatory cancer cell state was simply and rapidly simulated in vitro. 100 μg/mL NIN suppresses IL‐1β during inflammation, indicating its potential to mitigate the inflammatory response in colon cells. Conversely, at a dose of 200 μg/mL, NIN notably increases the expression of Muc2, which is typically reduced in inflammatory conditions. This result suggested that the higher concentrations of NIN could rejuvenate the mucin‐producing capability of colon cells under inflammatory stress. Further experiments demonstrated that these medium and high doses of NIN also significantly hinder the proliferation of HT‐29 cancer cells. These antiproliferative effects coincided with a marked reduction in the expression of Lgr5, a gene marker for colon cancer stem cells, suggesting NIN’s efficacy in targeting cellular mechanisms essential for tumor growth in inflammatory environments. Effects of NIN were inhibited by CH223191, an antagonist of the AhR signaling pathway. Decreased AhR signaling is supposed to contribute to amplifying the gut tissue’s destructive immune inflammatory responses. In the inflamed gut of IBD patients, AhR expression is defective [30]. AHR activation induces cytochrome P4501 (CYP1) enzymes, which have an important feedback role that curtails the duration of AHR signaling [31]. In our studies, the presence of NIN markedly escalates the expression of CYP1, indicating it is complicated of the impact of NIN on the AhR pathway. Considering the interaction between the AhR and Wnt signaling pathways, our studies found the presence of NIN also increase the expression of transmembrane E3 ubiquitin ligase ring finger protein (Rnf43), decreases the expression of the target gene Axin2 and the expression of the key molecule non‐phosphorylated β‐catenin, and then inhibits the Wnt signaling pathway which plays an antitumor role. The inhibition of the Wnt signaling pathway by NIN was blocked by the AhR antagonist CH223191. The results suggest that NIN might alleviate UC by activating AhR, possibly through the interaction between AhR and the Wnt signaling pathway, inhibiting the inflammatory cancer transformation.

Preliminarily, this study demonstrates that NIN could modulate inflammatory responses in cancer cells, enhance mucin secretion, and curtail the proliferation of colon cancer cells under inflammatory conditions. The perspective of inflammation‐cancer transformation sketchily explored the possible mechanism of NIN on UC. However, since UC‐associated colon cancer is not just an inflammatory state as in vitro. The limitations of our work may be expected to be broken through future research as follows:

a. The basal expression levels of AhR differ between normal and tumor environments. Normal cell groups and tumor cell groups can be comparatively established. NIN and its bioactive compounds can be used to act on the above two groups of cells, exploring the effects and dose–response relationships on AhR expression in different environments.
b. The same dose of AhR antagonist can antagonize the effects of low and medium doses of NIN, but some effects of high‐dose NIN are not inhibited. The reasons are speculated that: the antagonist at this dose is not sufficient to inhibit the effects of high‐dose NIN; high‐dose NIN may exert a synergistic effect with antagonists through the negative feedback regulatory mechanism of the AhR pathway. Further experiments are needed to explore this appearance.
c. In fact, the inflammatory transformation of colitis undergoes repeated inflammation‐healing processes, which can cause mutations in proliferation regulation‐related genes, leading to dysplasia and carcinogenesis. Based on the high content of active ingredients in NIN, this study only judges the potential effect of NIN on colitis‐related colon cancer at the in vitro level and elucidates its possible mechanism. Construction of a realistic colitis‐associated colon cancer model in vivo is essential to understand the link between inflammation and cancer development.

**Nomenclature**
AhR: Aryl hydrocarbon receptor
ANOVA: Analysis of variance
ARNT: AhR nuclear transporter
ATCC: American type culture collection
CAC: Colitis associated colorectal cancer
CCK‐8: Cell counting kit‐8
CON: Control group
CYP1: Cytochrome P450 family 1
DMSO: Dimethyl sulfoxideA
DRE: Dioxin‐responsive element
DSS: Dextran sulfate sodium salt
HPLC: High performance liquid chromatography
IBD: Inflammatory bowel disease
IN: Indigo naturalis
LPS: Lipopolysaccharide
MOD: Model group
NIN: Indigo naturalis prepared using a novel method
qRT‐PCR: Quantitative real‐time polymerasere chain reaction
SDS‐PAGE: Sodium dodecyl sulfate‐polyacrylamide gel electrophoresis
TCM: Traditional Chinese medicine
UC: Ulcerative colitis.

## Data Availability Statement

The data will be made available upon request.

## Conflicts of Interest

The authors declare no conflicts of interest.

## Author Contributions

Conceptualization: Xianxiang Xu and Yong Diao. Methodology: Xianxiang Xu and Xinyi Zhou. Investigation, Data curation: Lin Lin. Formal Analysis: Aftab Ullah. Writing – original draft): Lin Lin and Wenjie Ning. Writing – review and editing): Xianxiang Xu and Aftab Ullah. Supervision: Xiaoping Huang and Huiyong Yang. Administration: Xunxun Wu. Resources: Xinyi Zhou and Huiyong Yang. Funding acquisition: Yong Diao and Xianxiang Xu.

## Funding

The study was supported by the Key Project of Quanzhou City Science and Technology Program (Grant 2022N024), the National Natural Science Foundation of China (Grant U1405215), and the Promotion Program for Young and Middle‐aged Teacher in Science and Technology Research of Huaqiao University (Grant ZQN‐PY420).
